# Survival analysis and prognostic factors of timing of first childbirth among women in Nigeria

**DOI:** 10.1186/s12884-016-0895-y

**Published:** 2016-05-13

**Authors:** Adeniyi Francis Fagbamigbe, Erhabor Sunday Idemudia

**Affiliations:** 1 School of Research and Postgraduate Studies, Faculty of Human and Social Sciences, North West University, Mafikeng, South Africa; 2 Department of Epidemiology and Medical Statistics, Faculty of Public Health, College of Medicine, University of Ibadan, Ibadan, Nigeria

**Keywords:** Age at first birth, Education, Hazard ratio, Nigeria, Survival analysis

## Abstract

**Background:**

First childbirth in a woman’s life is one of the most important events in her life. It marks a turnaround when she might have to drop roles of career building and education, for motherhood and parenthood. The timing of the commencement of these roles affects the child bearing behavior of women as they progress in their reproductive ages. Prevalent early first childbirth in Nigeria has been reported as the main cause of high population growth and high  fertility, mortality and morbidity among women, but little has been documented on the progression into first birth as well as factors affecting it in Nigeria. This paper modelled timing of first birth among women in Nigeria and determined socio-demographic and other factors affecting its timing.

**Methods:**

We hypothesized that background characteristics of a woman will influence her progression into having first birth. We developed and fitted a survival analysis model to understand the timing of first birth among women in Nigeria using a national representative 2013 NDHS data. Women with no children were right censored as of the date of the survey. The Kaplan Meier survival function was used to estimate the probabilities of first birth not occurring until certain ages of women while Cox proportional hazard regression was used to model the timing of first births at 5 % significance level.

**Results:**

About 75.7 % of the respondents had given birth in the Northern region of Nigerian compared with 63.8 % in the South. Half (50.1 %) of the first childbirth occurred within the 15–19 years age bracket and 38.1 % within 20–29 years. The overall median survival time to first birth was 20 years (North 19, South 22), 27 years among women with higher education and 18 years for those with no formal education. The adjusted hazard of first birth was higher in the Northern region of Nigeria than in the South (aHR = 1.24, 95 % CI: 1.20-1.27), and higher in rural areas than in urban areas (aHR = 1.15, 95 % CI: 1.12-1.19). Also, hazard of earlier first birth tripled among women with no education (aHR = 3.36, 95 % CI: 3.17-3.55) compared to women with higher education. The significant factors affecting age at first birth are education, place and zone of residence, age at first marriage, religion, ethnicity and use of contraceptives.

**Conclusions:**

This study showed that progression into early first birth is most affected by the education standing of women as well as age at first marriage. Delay of first childbirths as a strategy for fertility reduction and maternal health improvement can be achieved if women are empowered early in life with quality education. Stakeholders should therefore, give adequate attention to educating the girl child. Adverse socio-cultural norms of betrothing and marrying young girls should be abrogated, while health education and promotion of need to delay child bearing must be intensified especially among rural dwellers and also in Northern Nigeria.

## Background

Age of initiation of childbearing among women has been reported to have strong effect on the demographic behavior of women and the entire population [1, 2]. The birth of the first child is a significant event that leaves social marks in the life of the woman. It is the obvious transition of a woman into parenthood with associated roles, responsibilities and deliverables. An early beginning of child birth may have adverse effect on a woman’s socio economic well-being in later years [1, 3, 4]. This is due to the fact that once child birth begins, the woman has to drop some roles to uptake other roles that may have great demands on her time and resources [5–7]. The adverse effects of this might be in the areas of career development, educational attainment, opportunity to marry in the future (if the birth is out of marriage), marital stability, asset possessions and most importantly her health. It also affects type of care and opportunity available to such women and their off-springs, social change, fertility trends and the state of the economy. Teenage pregnancies arising from early first birth have also been linked to maternal deaths [8–10]. These challenges could ultimately shape her future [11].

On the contrary, importance has been attached to delayed age at first child birth. While it reduces the rate of maternal and child morbidity and mortality on one hand, especially in Nigeria where the maternal mortality ratio (MMR) and infant mortality ratio (IMR) are above African Average [12]. On the other hand, delayed child bearing reduces fertility thereby, curtailing excessive population growth and improves standard of living among families through education and career development [6, 11, 13, 14].

**Fig. 1:**
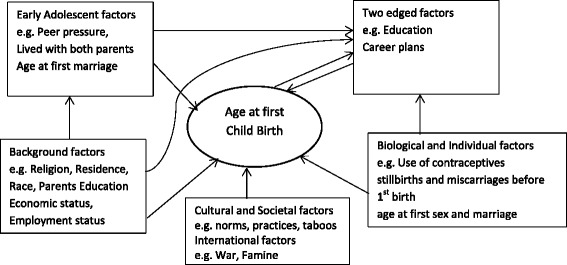
Theoretical relationship of socio-demographic and other factors with Age at first birth

Factors affecting age at first child are diverse and are of different levels. There are individual factors, family factors, societal factors as well as economic, national and international factors as illustrated in Fig. 1. The individual factors include personal attitudes such as, use of contraceptives and education. The family factors are made up of socio-economic background characteristics such as, education of parents, place of residence, geographical region of birth, religion, social status, employment status, etcetera. The societal factors include norms practices, peer pressures, women’s liberation and acceptance of cohabitation [6, 7, 11, 13–17].

### Theoretical considerations of the factors

#### Family background factors

The most prominent among the influencing factors are the individual and family background. In this study, we hypothesized that individual and family characteristics would influence the timing of first childbirth among women. On educational attainment, it is not unlikely that girls who could not progress in their educational pursuit would consequently start child bearing earlier than others. Thus, girls from the northern part of the country where literacy level has been put at 22 % compared with 80 % in the south [1, 10] might be more exposed to child bearing earlier than those from the south. Religion affiliation might affect age at first birth. For instance, the Catholics forbid use of contraceptives apart from the regular rhythm, although the doctrine emphasized abstinence outside marriage. In Nigeria, the scenario can be argued in two ways: firstly, the Muslim dominated north where early marriage is more prevalent may have early age at first birth than others, in the opposite; secondly, the Catholic dominated south may also have early age at first birth due to non-use of contraceptives.

Rural area is about a two thirds of Nigeria landscape and contributes about a third of the population [1, 18]; this may seriously affect age at first birth because literature is replete with the fact that women raised in rural areas become mothers at a much younger age than those brought up in urban areas [1]. In rural settings, where opportunities such as school enrollment and job opportunities are very scarce, early parenting may be a welcome alternative. Another peculiarity in rural areas is farming, which requires little or no training with virtually no initial capital in Nigeria. So the urge to postpone births till a later age does not arise among youths, since they often start childbearing as soon as they joined their parents in farming or when they start on their own.

### Early adolescent factors

Studies have identified peer pressure as one of the factors shaping behavior and attitude of adolescents [3, 15, 16]. Often, some peer groups pressurize young girls to partaking in activities such as alcohol drinking and cigarette smoking that could probably increase their chance of entry into early motherhood. They therefore, tend to begin “adult” activities such as sexual intercourse early, leading to marriage and ultimately result in irrevocable entry into child bearing [3, 5, 13, 19].

### Biological factors

Ability of females to get children might affect the age at which they begin parenting. Number of miscarriages and stillbirths before first birth can be used as a proxy to how fecund a woman is. A less fecund woman would ultimately have a later age at first birth than those that had no miscarriage before first birth [3, 15, 20]. Also, correct use of reliable contraceptive methods might delay child bearing. We expect women using contraceptives to have a late first child birth than those who do not use contraceptives. Similarly, marriage has been reported to be positively correlated to early first childbirth [21, 22].

### Social and cultural factors

Cultural practices such as betrothing girls right from birth or as soon as they are about ten years old to a would be husband [10, 21], can adversely affect timing of first childbirth. This may not be untrue in Northern Nigeria where the practice is common and the age at first birth is much lower than in the South [1, 23]. Conversely, inability to find a suitable person to marry or inability to pay huge marriage rites, as practiced in certain parts of Nigeria, may elongate the age at first marriage [10, 21, 24] and by extension, the age at first birth.

### Two edged factors

#### Education and employment

Education and employment have dual way relationship with age at first birth. Whether decisions by teenagers on entrance into motherhood are deliberate or not, it has consequential effects on their educational attainment and employability. It is extremely difficult and very rare to combine teenage motherhood with education. While determination to acquire higher education might lead to postponement of first child birth on one hand, early first child birth might stop, or at least temporarily halt, educational process on the other hand [4, 11, 13, 16, 19, 25]. Entrance into labor market before the first child birth could also have a reciprocal association with age at first childbirth. Women who work before first birth often delay the birth so as to work longer. Meanwhile, women who began childbearing too early without any career development may find themselves unemployable in the future [17, 26].

Effect of age at marriage may be very significant on fertility and the age at which women began childbearing [17] as it is a proxy for beginning of a woman’s exposure to risk of pregnancy. However, it may have a limited influence on the age at which a woman begins child bearing, if it began irrespective of whether one has been married or not. Moreover, fecundity is related to women’s age and not to age at marriage [3].

Globally, age at first child birth is been delayed [4, 5, 27]; increase in age at first birth has been associated with reduced fertility rates in some countries [28]. For instance, in Italy between 1980 and 1996, the mean age at first birth which increased from 25.0 years to 28.4 years was accompanied by a decline of Total fertility Rate (TFR) from 1.64 to 1.19 [27]. These delays have been linked to female taking more educational opportunities, labor market participation and also because of difficulties in combining child bearing with education and employment [15, 26, 29].

While developed countries such as the USA have consistently reported high and increasing age at first birth, rising from 22.6 to 25.3 years, a 12 % increase between 1980 to 2010 [19]. In contrary, a woman in Nigeria would have had her first birth 6 years earlier than a girl in the USA on the average [1, 23]. Although previous studies have documented factors affecting first birth, literature is however, limited on monitoring the timing of first birth from when a woman is exposed to child bearing to the time of first child birth without omitting those who have not experienced a child birth in the analysis as they are also at the risk of child birth. This study is therefore, designed to assess progression into first births and model timing of first child birth among Nigeria women. In this study, we carried out a retrospective follow up study of first birth timings viz-a-viz the socio-demographic and cultural characteristics of women. We determined the significant risk factors and provided evidence based recommendations to stakeholders so that informed decisions could be taken. This will help curtail early first births among Nigerian women so that remote and long term side effects of early first child birth could be abated. This study is of high significance because its outcomes will answer questions on how to delay first birth and by extension reduce the prevalent high fertility in Nigeria.

## Methods

### Study design and setting

Data from the 2013 Nigeria Demographic and Health Survey (NDHS) [1], a cross-sectional national representative data, was used for this study. The National Population Commission (Nigeria) and ICF International, United States gave access and authorized the use of the data. The survey used clusters as the primary sampling unit based on the EAs from the 2006 census frame and sampled respondents using a stratified three-stage cluster design consisting of 904 clusters, 372 in urban areas and 532 in rural areas across the six zones, 36 states, and the Federal Capital Territory, Abuja. A total of 39,902 women aged 15–49 years were identified as eligible for individual interviews, and 98 % of them were successfully interviewed.

We extracted information on the women’s background characteristics, sexual and reproductive history and knowledge, source and use of contraception. The dependent variable in this study was age at first child birth while region and geographical zones of residence, education, religion, residence and ethnicity were the independent variables. Included also as independent variables are responses on; if the woman ever smoked, whether she had terminated a pregnancy or not and whether she has ever used something to prevent pregnancy. We collapsed the six zones into two regions: The North Central, North East and North West constituted the “North” while South East, South South and South West formed the “South”. We used women’s current educational attainment as a proxy for education as of the time of first child birth. This is justified, because the educational status does not change for persons who had none or primary education throughout their lifetime since primary education is mostly attained at age 12. “Ever smoked” was used as a proxy for peer pressure. We used survival analysis to model the determinants of age at first birth.

### Rationale for use of survival analysis

Survival analysis is analysis of history of events which uses statistical procedures to deal with analysis of time duration, until one or more events of interest happen. It is usual in a follow up study, such as the current study, for some participants not to have experienced the event of interest at the end of the study or some participants were “lost to follow up” or some might have withdrawn during the study. Bias may be introduced if these categories of participants were excluded in further analysis as they could possess unique characteristics that could be useful in answering the research question. In such cases, the length of time the participants stayed in the study would be recorded as their study time and marked as “censored”.

Two quantitative terms are important in survival analysis. They are the survivor function *S*(*t*) and hazard function *h*(*t*). In relation to the present study, the survivor function gives the probability that a woman “survives” longer than some specified time *t* without a birth, while the hazard function gives the instantaneous potential per unit time to have a first childbirth after time *t*, given that the individual had not had a first childbirth up to time *t*. Survival and hazard function are mathematically denoted by

1 $$ s(t)={S}^{\prime }(t)=\frac{d}{dt}S(t)=\frac{d}{dt}{\displaystyle {\int}_t^{\infty }f(u)du=\frac{d}{dt}\left[1-F(t)\right]}=-f(t). $$

and

2 $$ \lambda (t)=\begin{array}{c}\kern1em  \lim \kern1em \\ {}{\kern1em }^{dt\to 0}\kern1em \end{array}\frac{ \Pr \left(t\le T<t+dt\right)}{dt\left(S(t)\right)}=\frac{f(t)}{S(t)}=-\frac{S^{\prime }(t)}{S(t)} $$

In contrast to the survivor function (*S*(*t*)) which describes the probability of not failing before time *t*, hazard function (*h*(*t*)) addresses the failure rate at time t among those individuals who are alive at time *t*. Also two variables are compulsory in survival analysis; they are survival time and the censoring index. The “survival time” or “follow-up time”, is assumed to be a discrete random variable that takes on only positive integer.

In this study, the population at risk are all women involved in the study since they are all likely to give birth one time or the other. The “survival time” for age at first childbirth is the age of the women at first birth while the survival time for those with no birth as of the time of the survey was their current age at the time of the survey. Thus their censoring index were coded “1” and “0” respectively. The usual logistic regression techniques therefore, become unsuitable in a follow up study such as the current study where the follow up time could be determined and used in explaining the event of interest.

Kaplan-Meier method, developed for scenarios where survival time is measured on a continuous scale whereby only intervals containing an event contribute to the estimate, was used to compute the survival estimates. The Kaplan-Meier estimates of *S*(*t*) were obtained from equation (3) where *n*_*j*_ is the number of subject observed at time *tj* and *dj* is the number of subject that experienced the event of interest at time *tj*.

3 $$ s(t)={\prod}_{j=1}^k\frac{\left(nj-dj\right)}{nj} $$

We applied the Cox-proportional Hazard model to the age at first birth. The model assumed that proportion of hazards are constant from time to time. In proportional hazard model, the effect of a unit increase in covariate is multiplicative with respect to hazard rate. The Cox model gives an expression for the hazard at time *t* for an individual with a given specification of a set of independent variables denoted by *X* to predict individuals’ hazard. The model assumes the relationship for one covariate where h_o_(t) is the baseline hazard function, xi are the covariates and *β*_*i*_ are the coefficients.

4 $$ h\left(t;x\right)={h}_0(t) \exp \left(x\beta \right) $$

We also determined Cox regression estimates for all levels of each of the covariates. In which case, the hazard at time *t* for a subject in group is assumed to be

5 $$ {h}_i(t)={h}_{i0}(t) \exp \left({\beta}_1{x}_{i1}+\dots +{\beta}_k{x}_{ik}\right) $$

The coefficients are assumed to be the same, regardless of group, but the baseline hazard can be group specific. The sign of the coefficient indicates how a covariate affects the hazard rate. The hazard ratio (HR), expressed as the exponentials of the coefficients, implies more exposure to event of interest if >1, HR < 1 means low exposure while HR = 1 has no effect on the exposure. The statistical significance of the coefficient indicates whether these changes in the expected duration will be statistically significant or not. In the stratified Cox analysis, we tested whether the proportional-hazards assumption was violated using the significance of the hazard ratios, the log likelihood tests and the Wald chi square statistics.

The significant variables in the independent Cox regression were plugged into the multiple Cox regression so as to control for the effects of other variables. We fitted four models. The first model involved the two most significant independent variables at the bivariate logistic level, Model II involved the other socio-cultural characteristics of the women (place and regions of residence, ethnicity and religion). In model III we added education and age at marriage to the socio-cultural factors while model IV is the full model. The log likelihood tests and the Wald chi square statistics were used to select the best model. The data was weighted to adjust for differences in population in each state and FCT. Statistical significance was determined at *p*-value = 0.05. We used the Stata (version 13) statistical analysis software for all the analysis.

## Results

About 70.9 % of the 38,948 respondents had given birth to at least a child, 75.7 % in the North compared with 63.8 % in the South, 86.4 % among women with no education and 55.3 % among women with higher Education. Half (50.1 %) had their first childbirth within the 15 to 19 years age bracket, 38.1 % within age 20–29 years, 9.0 % before attaining age 15 and 2.8 % at 30 years or later. Almost 70 % of Northern women had given birth before age 20 years compared with 43.0 % in the South as shown in Table 1. All variables considered were significantly associated to timing of first child birth.

**Table 1:** Distribution of respondents by characteristics, birth history and summaries of the age at first childbirths

		Ever given Birth	Timing of first Birth (years) among “Had Birth”				Median (years)	Mean ± σ (years)
Characteristics	*N*		<15	15–19	20–29	30+		
Region								
North Central	6251	4190(68.9)	^a^271(6.5)	1866(44.5)	1913(45.7)	140(3.3)	19	20.0 ± 4.0
North East	6630	5096(76.1)	623(12.2)	3018(59.2)	1387(27.2)	68(1.3)	17	18.2 ± 3.7
North West	9673	7730(78.7)	1012(13.1)	4945(64)	1717(22.2)	56(0.7)	17	17.7 ± 3.4
South East	4462	2621(57.6)	140(5.3)	881(33.6)	1425(54.4)	175(6.7)	21	21.5 ± 5.0
South South	6058	3830(62.4)	282(7.4)	1796(46.9)	1620(42.3)	132(3.5)	19	19.9 ± 4.4
South West	5874	3984(69.3)	130(3.3)	1253(31.5)	2394(60.1)	207(5.2)	21	21.6 ± 4.4
Residence								
Urban	15545	9778(63.4)	^a^545(5.6)	3844(39.3)	4944(50.6)	445(4.6)	20	20.7 ± 4.6
Rural	23403	17673(76.4)	1913(10.8)	9915(56.1)	5512(31.2)	333(1.9)	18	18.6 ± 4.0
Education								
education	13740	11952(86.4)	^a^1557(13)	7292(61.0)	2956(24.7)	147(1.2)	17	17.9 ± 3.7
Primary	7104	5953(83.4)	548(9.2)	3156(53.0)	2125(35.7)	124(2.1)	18	19.0 ± 4.0
Secondary	14407	7475(52.5)	308(4.1)	2964(39.7)	3958(53.0)	245(3.3)	20	20.7 ± 4.2
Higher	3697	2071(55.3)	45(2.2)	347(16.8)	1417(68.4)	262(12.7)	24	24.0 ± 4.9
Age at first Marriage								
Never	9326	617(6.6)	50(8.1)	313(51.0)	239(38.9)	12(2.0)	19	19.2 ± 3.9
Before 15	7869	7396(94.0)	2148(29.2)	4565(62.1)	604(8.2)	35(0.5)	15	15.8 ± 2.8
15–19	13187	11925(90.4)	203(1.7)	8450(71.3)	3150(26.6)	51(0.4)	18	18.4 ± 2.4
20+	8566	7677(89.6)	78(1.0)	488(6.4)	6376(83.6)	689(9.0)	23	24.0 ± 4.0
Religion								
Catholics	4081	2409(58.3)	^a^138(5.7)	937(38.9)	1207(50.1)	127(5.3)	20	20.9 ± 4.8
Other Christian	15757	10107(64.4)	646(6.4)	4030(39.9)	4972(49.2)	459(4.5)	20	20.6 ± 4.6
Islam	18578	14504(77.9)	1617(11.2)	8592(59.2)	4112(28.4)	183(1.3)	17	18.3 ± 3.8
Others	532	431(79.1)	57(13.2)	200(46.4)	165(38.3)	9(2.1)	18	18.9 ± 4.3
Ethnicity								
Yoruba	5606	3710(67.9)	^a^107(2.9)	1108(29.9)	2315(62.4)	180(4.9)	21	21.7 ± 4.3
Igbo/Ibiobio	5448	3202(58.5)	151(4.7)	1044(32.6)	1776(55.5)	231(7.2)	21	21.7 ± 5.0
Hausa/Fulani	11811	9524(79.9)	1223(12.8)	6092(64.0)	2120(22.3)	89(0.9)	17	17.7 ± 3.5
Others	16083	11015(68.6)	977(8.9)	5515(50.1)	4245(38.5)	278(2.5)	19	19.3 ± 4.2
Ever Smoked								
No	38796	27327(70.9)	^a^2440(8.9)	13703(50.1)	10412(38.1)	772(2.8)	19	19.4 ± 4.4
Yes	152	124(78.9)	18(14.5)	56(45.2)	44(35.5)	6(4.8)	18	19.1 ± 4.8
Ever Had pregnancy terminated								
No	29528	20235(68.9)	^a^2156(10.2)	12049(54.3)	9026(33.1)	627(2.4)	18	19.3 ± 4.3
Yes	9420	7216(87.2)	302(5.5)	1710(38.4)	1430(52)	151(4.1)	19	19.7 ± 4.7
Ever Used Contraceptives								
No	34834	23858(68.9)	^a^2063(9)	10988(50.5)	6701(37.8)	483(2.6)	18	18.9 ± 4.2
Yes	4114	3593(77.2)	395(8.4)	2771(47.6)	3755(39.8)	295(4.2)	20	20.7 ± 4.5
Region								
South	16394	10435(63.8)	^a^552(5.3)	3930(37.7)	5439(52.1)	514(4.9)	20	20.9 ± 4.6
North	22554	17016(75.7)	1906(11.2)	9829(57.8)	5017(29.5)	264(1.6)	18	18.4 ± 3.9
Total	38948	27451(70.9)	2458(9)	13759(50.1)	10456(38.1)	778(2.8)	19	19.4 ± 4.4

^a^ Significant at 5 % *X* ^*2*^ statistics

**Fig. 2:**
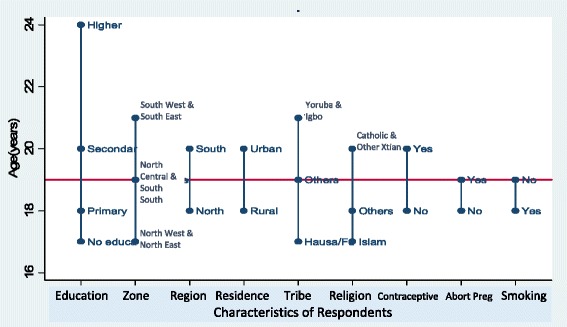
Distribution of Median age at first births of the respondents’ by their characteristics

The overall median age at first birth was 19 years, 18 years in the North and 20 years in the South; 20 years in urban areas and 18 years in the rural areas; 17 years among Hausa/Fulanis and Muslims compared with 21 years among Yorubas and Igbos. The median age at first birth was 24 years among women with higher education compared with 17 years, among those with no education (Table 1 and Fig. 2).

The overall Median Survival Time (MST) was 20 years, with 19 years in the Northern region and 22 years in the south. Also, MST was 27 years among women with higher education and 18 years among those who never had education, 24 years in the South East and 18 years in each of North West and North East. The overall incidence rate (IR), technically defined as the probability that an event will occur at time *t*_*k+1*_ given that it did not occur at time *t*_*k*_ *,* is 0.036. It is the probability of birth occurring at a time (year) given that the woman has had no child before that time. The IR was highest (0.048) among women with no education, who married before age 15 (0.059), from North West (0.045), Hausa/Fulani (0.045), had only primary education (0.044) and lives in North East (0.041). The hazard of early first birth was higher in all zones than in the South West except in the South East. The hazard of first birth in North East and North West was 1.86(95 % CI: 1.79-1.94) and 2.26(95 % CI: 2.17-2.35) respectively, higher than hazard of their counterparts from the South West. The hazard of early first birth in rural areas almost doubled, 1.72(95 % CI:1.68-1.76), the hazard among those from urban areas. Also, hazard of first birth among women with no education was over four times higher, 4.43(95 % CI: 4.22-4.64) than among women with higher education as shown in Table 2.

**Table 2:** Median survival times and unadjusted hazard ratio of timing of first births among Nigerian women

Characteristics	MST (years)	Incidence rate	Hazard ratio	95 % CI of HR	*p*-value
Region					
North Central	21	0.033	1.18	*1.13–1.23	0.000
North East	18	0.041	1.86	*1.79–1.94	0.000
North West	18	0.045	2.26	*2.17–2.35	0.000
South East	24	0.027	0.80	*0.77–0.85	0.000
South South	21	0.031	1.11	*1.06–1.16	0.000
South West^a^	22	0.032			
Residence					
Urban^a^	22	0.030			
Rural	19	0.040	1.72	*1.68–1.76	0.000
Education					
None	18	0.048	4.43	*4.22–4.64	0.000
Primary	19	0.044	3.43	*3.26–3.61	0.000
Secondary	22	0.026	1.81	*1.72–1.90	0.000
Higher^a^	27	0.023			
Age at 1^st^ Marriage					
Never	na	0.003	0.18	*0.17–0.20	0.000
Before 15	15	0.059	6.12	*5.92–6.33	0.000
15–19	18	0.049	3.07	*2.98–3.17	0.000
20+	24	0.037			
Religion					
Catholics^a^	23	0.028			
Other Christians	22	0.031	1.17	*1.12–1.23	0.000
Islam	18	0.042	2.21	*2.12–2.31	0.000
Others	19	0.042	1.93	*1.75–2.14	0.000
Ethnicity					
Yoruba^a^	23	0.031			
Igbo/Ibiobio	24	0.027	0.80	*0.76–0.83	0.000
Hausa/Fulani	18	0.045	2.33	*2.24–2.42	0.000
Others	20	0.035	1.34	*1.29–1.39	0.000
Smoking					
No	20	0.036			
Yes^a^	19	0.040	1.05	0.88–1.26	0.553
Ever Had pregnancy terminated					
No^a^	20	0.035			
Yes	20	0.043	1.08	*1.04–1.12	0.000
Ever Used Contraceptives					
No	19	0.036	1.31	*1.27–1.34	0.000
Yes^a^	22	0.036			
Region					
South^a^	22	0.030			
North	19	0.040	1.78	*1.74–1.82	0.000
Total	20	0.035			

^a^Reference category *significant at *p* < 0.05 MST Median Survival Time

**Fig. 3:**
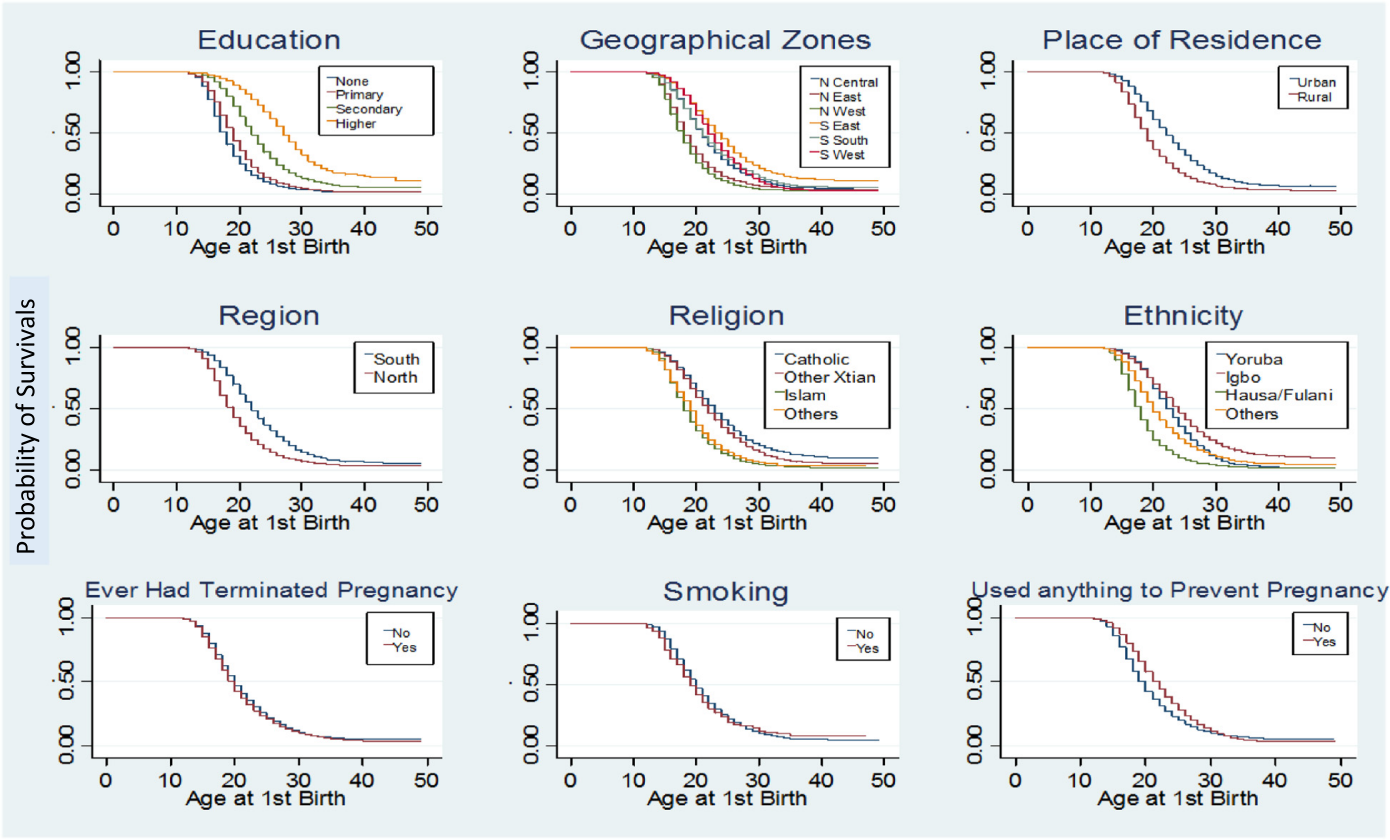
Survival functions for patterns of Timing at first birth by respondents characteristics

The survival functions of the age at time of first childbirth by respondents’ characteristics are shown in Fig. 3. It shows the probability that first childbirth has occurred at age t. For instance, the probabilities of having a first child at various ages as a woman progresses into adulthood is consistently lower among those that had higher education than others with lesser educational attainment. The likelihood Chi square test and the Wald’s test showed significant differences in the survival function of all the factors studied except across smoking status.

In Table 3, we presented the result of multiple Cox-proportional hazard models of relationship between timing of first childbirth and independent variables. Four models were fitted for the survival data. Model I was restricted to educational attainment and region. This was aimed at testing if only education and region of residence predicts timing of first childbirth. There were higher hazards of early first child in the North (aHR = 1.24; *p* < .05; model I) and among those with no education (aHR = 3.97; *p* < .05; model I) and primary education (aHR = 3.39; *p* < .05; model I). Model II contained only demographic characteristics without education; this was to see possibility of having significant factors predicting age at first childbirth besides education. Hazards of age at first childbirth were higher in North East (aHR = 1.14; *p* < .05; model II) and North West (aHR = 1.21; *p* < .05; model II) than in South West but lower in North Central and South South. Hazard of first child birth was higher (aHR = 1.42; *p* < .05; model II) than in urban area and higher (aHR = 1.37; *p* < .05; model II) among the Hausa/Fulanis, than any other tribe.

**Table 3:** Multiple cox proportional hazard models of relationship between timing of first childbirth and background characteristics

	Model I	Model II	Model III	Model IV
Characteristics	aHR(95 % CI)	aHR(95 % CI)	aHR(95 % CI)	aHR(95 % CI)
Residence				
North Central		*0.89(0.84–0.94)	0.96(0.91–1.02)	0.99(0.94–1.05)
North East		*1.14(1.07–1.21)	*1.16(1.09–1.23)	*1.20(1.13–1.28)
North West		*1.21(1.13–1.29)	*1.22(1.14–1.31)	*1.26(1.18–1.35)
South East		1.00(0.91–1.09)	0.95(0.87–1.03)	0.98(0.90–1.07)
South South		*0.94(0.88–1.00)	1.03(0.97–1.09)	1.02(0.96–1.08)
South West^a^				
Residence				
Urban^a^				
Rural		*1.42(1.38–1.46)	*1.14(1.11–1.17)	*1.15(1.12–1.19)
Education				
No education	*3.97(3.77–4.17)		*3.17(3.00–3.36)	*3.36(3.17–3.55)
Primary	*3.39(3.22–3.57)		*3.12(2.96–3.29)	*3.24(3.08–3.42)
Secondary	*1.83(1.74–1.92)		*1.75(1.67–1.84)	*1.79(1.71–1.88)
Higher^a^				
Age at 1^st^ Marriage				
Never^a^			*0.16(0.14–0.19)	*0.19(0.18–0.20)
Before 15			*5.92(5.57–6.01)	*6.31(5.92–6.56)
15–19			*3.00(2.89–3.10)	*3.02(2.92–3.13)
20+				
Religion				
Catholics^a^				
Other Christian		*1.07(1.02–1.12)	*1.07(1.02–1.12)	*1.08(1.03–1.13)
Islam		*1.43(1.35–1.51)	*1.12(1.06–1.19)	*1.16(1.10–1.23)
Others		*1.54(1.39–1.71)	*1.16(1.05–1.29)	*1.21(1.09–1.35)
Ethnicity				
Yoruba^a^				
Igbo/Ibiobio		*0.86(0.80–0.94)	*0.83(0.77–0.91)	*0.85(0.78–0.93)
Hausa/Fulani		*1.37(1.28–1.46)	*1.11(1.04–1.18)	*1.16(1.09–1.24)
Others		*1.18(1.11–1.24)	0.99(0.93–1.04)	1.02(0.96–1.08)
Ever Had pregnancy terminated				
No^a^				
Yes				*1.09(1.05–1.13)
Ever used contraceptives				
No				*0.79(0.76–0.81)
Yes^a^				
Region				
South^a^				
North	*1.24(1.20–1.27)			
−2*Log* L	526,294.41	528,314.50	525,688.86	525,434.08
Chi Square	7029.51	5009.11	7634.74	7889.53

aHR adjusted Hazard ratio CI Confidence Interval ^a^ Reference *Significant at *p* < 0.05

In Model III, we introduced education and age at first marriage that were not included in Model II. Inclusion of Education and age at first marriage in the Model increased the hazard of first birth across geopolitical zones, lowered the hazard in rural areas and across ethnicity and religion affiliations. Model IV was the full Model containing all variables that was earlier dropped due to insignificance in the independent Models. The hazards reduced generally across the factors considered in the full model. For instance, the hazard among women with no education reduced from 4.43 to 3.36. The hazard in North West was 1.26; *p* < .05; model IV and North East was 1.26; *p* < .05; model IV compared with South West. Hazard of early first birth was higher in the rural areas (aHR = 1.15; *p* < .05; model IV)) compared with women in Urban areas and but lower aHR = 0.79; *p* < .05; model IV) among women who have never used anything to prevent pregnancy compared with those who did. Similarly, women who had ever had pregnancy terminated had higher hazard (aHR = 1.09; *p* < .05; model IV) than those who never did. The full Model (Model IV) was the best model as it had the best statistics (Table 3).

## Discussion

In this study, we sought to monitor and describe timing of first childbirth among women in Nigeria and modeled factors affecting it using survival analysis techniques. We found that early first childbirth is prevalent in the studied population and identified educational attainment and age at first marriage as the most significant determinant of age at first child birth. This finding was in concurrence with the outcome of a Vietnam study which reported that majority of the women in the study had their first birth before age 20 [7] and 15 years reported in a Bangladesh study [3] but much lower than the median ages in most developed countries [4, 19, 25]. However, another Bangladesh study had affirmed that 72.8 % women had first childbirth before attaining age 20 years with an average of 18.7 years as age at first birth [13].

We found educational attainment to be the strongest risk factor of age at first birth in Nigeria, unlike the outcome of a Bangladesh study which reported family pressure as the most significant factors compared with other socioeconomic and cultural factors [13]. The full model in our study suggested that women with no education or primary education had higher hazards of having early first childbirth than those with higher education. This finding was in concurrence with the outcome of a Vietnam study which found women with secondary education to have a significantly higher age at first birth than those with little or no education [7] and also elsewhere [5, 13, 19, 28]. This is very understandable since women who choose to be educated would most likely postpone child bearing while, those who started child bearing early would have truncated educational ambition. It is not unlikely that postponement of first childbirth is connected with increasing proportions of women having higher education among Nigeria women [1, 23, 30]. This finding suggests that policies must emphasize education of the girl child.

Age at first childbirth was also positively associated with age at first marriage. Persons who married much early had first child birth earlier than those who delayed marriage. There might have been an interplay between age at first marriage and educational attainment as delaying marriage might give room for better education. This is an indicator that policies must be put in place to ensure better education for girls so as to delay marriages and thereby reduce fertility and other ills associated with early-age pregnancies and childbirths.

Postponement of first childbirth was associated with where a woman lives. This finding was in agreement with existing literature. Generally, women in Northern Nigeria have been reported earlier to be more likely to marry early and thus have early childbirth than their counterparts in the South [21, 24, 31]. In the same vein, we found women in the North West and North East to be more prone to early first birth compared with women in South West and South East zones. Similar patterns have been reported earlier [1, 23].

There was also rural–urban differentials in the timing of first births in Nigeria. Women living in rural areas have higher likelihood of early child birth than women in the urban areas. This is in consonance with the findings of a Vietnam study that found women from the North to have significantly higher age at first birth than women from the South [7]. Literature has consistently agreed that geographical variations exist in timing of first births [3, 7, 13, 15, 20, 32–34]. The availability of better infrastructures and opportunities in urban area could have affected this disparity. For instance, it is easier for an urban girl to get a job or gain admissions for further education than a rural girl which might serve as catalyst for delaying first birth. In addition, there could be more parents with better education in the urban than in the rural areas which could have favored urban girls in terms of guidance, motivation and mentoring. In such a situation, rural girls may be forced to result to early child bearing.

There were differences in age at first births along religious affiliation and ethnicity. Women practicing Islam were more likely to have earlier first birth than those affiliated to Catholic or other Christian denominations as reported earlier [23]. Similar findings have been documented in Bangladesh; women in Islamic religion had a tendency of early first birth than women in other religions [3]. In a similar pattern, women of Hausa/Fulani ethnic group had shorter time to first birth than the Yoruba and Igbo women.

The natural arrangement of Nigeria settlements can explain the apparent variations in her reproductive behavior. The North is dominated by the Hausa/Fulani who are mostly Muslims, while the Southerners are either Yoruba or Igbos and majority practices one Christian denomination or the other. Although we did not find multicollinearity among these variables, Nigerian women of Hausa/Fulani ethnic group practicing Islam Religion and living in the North, were more likely to have first birth earlier than any other group of women. This is coupled with the fact that the Northern zones have highest illiteracy levels in the country [1, 9, 10, 23, 30]. In essence, a Northern Nigerian woman would most likely have a great-grandchild at 50 years going by average of 17 years per cycle. This scenario might produce close generations of mothers, children parenting children and it endangers their health and overall well been of the populace. This shorter timing of first child birth could explain why the maternal and infant health indicators are worse in the North than in the South [1, 23, 30].

We found women who had ever used anything to prevent pregnancy to have delayed first birth and thus have lower likelihood of early first birth than those who never did. This could be explained by the fact that those who used something to prevent pregnancy were in control of their fertility and chose to postpone child bearing. This was in agreement with previous studies [3, 5]. Furthermore, literature has documented lower knowledge and use of contraceptives among the women in the North of Nigeria than in the South [1, 23, 30]. Although there was no significant difference in the median age, as well as median survival age at first birth between those who had ever had any pregnancy terminated or not, our fitted Cox proportional hazard model suggested a higher rate of early first child birth among those who ever had pregnancy terminated than those who did not. Smoking as a proxy for peer pressure was not significant to age at first birth in this study.

A key strength of this study is the use of a nationally representative and population-based data to model the timing of first births in Nigeria. However, the data used for this study might have suffered accuracy due to recall bias since data was self-reported and there were no alternative means of verifying the provided information. In addition, using secondary data limited the researchers in the choice of variables included in the analysis.

## Conclusions

The age at first birth is lower in Nigeria in comparison to the global average and developed countries. In Nigeria, early first child births have been mostly influenced by education and age at first marriage. Women who married before age 15 and who had no education or only primary education were the worst hit. Living in the Northern part of Nigeria where women are less educated, lack good knowledge and use of contraceptives lowered age at first birth. The early timing of first birth in Nigeria might have contributed to the high total fertility rate of 5.7 compared with less than 2.0 in Western Europe and the over 550/100000 maternal mortality rate and about 69/1000 infant mortality ratio and other poor maternal outcomes.

It is imperative to postpone the timing of first birth in order to harvest the multidimensional benefits accruing not only to the mother and her child but also to her family and the population at large. While we are aware that it may be difficult to delay first birth in the complex sociocultural setting presented in Nigeria, policies must be put in place to ensure meaningful education for the girl child. Adverse socio-cultural norms and practices that encourages early marriage and betrothing young girls should be abrogated, while health education and promotion on use of contraceptives and the need to delay child bearing must be intensified.

### Ethical approval and consent to participate

Data from the 2013 Nigeria Demographic and Health Survey (NDHS) [1], a nationally representative was used for this study. The National Population Commission (Nigeria) and ICF International, United States gave access and authorized the use of the data. Ethical approval for the study was sought and obtained from the National Health Research Ethics Committee assigned number NHREC/01/01/2012 as earlier documented [1]. All participants gave written and verbal informed consent prior to their enrollment into the study.

### Availability of data and materials

The data used for this study is readily available with DHS Measure.
